# 产科相关弥散性血管内凝血1例报告并文献复习

**DOI:** 10.3760/cma.j.cn121090-20241129-00498

**Published:** 2024-12

**Authors:** 闫芳菲 宋, 晓莹 龚, 彬 臧

**Affiliations:** 中国医科大学附属盛京医院重症医学科，沈阳 110004 Department of Critical Care Medicine, Shengjing Hospital of China Medical University, Shenyang 110004, China

**Keywords:** 产后出血, 弥散性血管内凝血, 治疗, Postpartum hemorrhage, Disseminated intravascular coagulation, Treatment

## Abstract

**目的:**

报道1例产科相关弥散性血管内凝血（disseminated intravascular coagulation，DIC）的患者，并讨论此类病例的诊疗方案。

**方法:**

对1例羊水栓塞后心脏骤停并发DIC患者的临床资料和治疗过程予以整理分析，并复习相关文献。

**结果:**

患者36岁，初产妇，因“停经9个月余、下腹痛14 h伴阴道少量流血2 h”入院待产，产后发生羊水栓塞，出现心脏骤停及DIC，继而导致大量失血，经积极循环呼吸支持、先后2次手术干预、输注红细胞、血浆、冷沉淀、纤维蛋白原等血液制品替代及早期抗纤溶等综合治疗后成功挽救患者生命，无不可逆的器官功能损害发生。

**结论:**

对产科相关DIC的患者，除治疗原发病外，还需通过成分输血替代治疗、酌情抗纤溶等纠正凝血功能，以改善预后。

## Abstract (en)

**Objective:**

To report a case of obstetrical disseminated intravascular coagulation and discuss the treatment strategy of such cases.

**Methods:**

The clinical data and treatment process of the patient with cardiac arrest and disseminated intravascular coagulation after amniotic fluid embolism was analyzed, and the related literature was reviewed.

**Results:**

Female, 36 years old, primigravida, was admitted to the hospital for delivery because of “amenorrhea for more than 9 months, lower abdominal pain for 14 hours with a small amount of vaginal bleeding for 2 hours”, amniotic fluid embolism occurred after delivery, then cardiac arrest and DIC happened, which led to a large amount of blood loss, through the treatment methods of active circulatory and respiratory support, surgical interventions for two times, transfusion of erythrocytes, plasma, and cold precipitation, fibrinogen and other blood product for substitution, and early antifibrinolytic, the patient's life was successfully saved without irreversible organ function damage.

**Conclusion:**

For patients with obstetrical disseminated intravascular coagulation, in addition to treating the primary disease, it is necessary to correct coagulation function by component transfuse and antifibrinolytic as appropriate to improve the prognosis.

弥散性血管内凝血（disseminated intravascular coagulation，DIC）是微血管体系被致病因素损伤后凝血活化，导致全身微血栓形成、凝血物质大量消耗并继发纤溶亢进，引起以出血及微循环衰竭为特点的临床综合征[1]。多种疾病可诱发DIC，如产科并发症、白血病、脓毒症及创伤等。本文报告了1例羊水栓塞诱发DIC患者的诊治过程，分享如下。

## 病例资料

患者36岁，初产妇，因“停经9个月余，下腹痛14 h伴阴道少量流血2 h”2020年5月6日就诊于我院。末次月经：2019年8月10日，停经42 d行超声检查提示宫内妊娠，自诉本次孕期定期于外院产检，未见异常。孕3产0，人流2次，2019年因宫腔粘连于外院行手术治疗。入院时血常规、凝血项等检查未见异常，入院诊断：①先兆临产；②孕3产0妊娠38周+4天，左枕前位；③高龄初产。2020年5月6日22:10患者出现规律宫缩，内诊宫口开大3 cm，送入分娩室待产，5月7日5:15侧切分娩一活婴。5:42胎盘仍未自行娩出，5:45手取胎盘困难，胎盘胎膜破碎取出，胎盘粘连植入不排除。阴道流血多，予卡贝缩宫素100 µg静滴、子宫按摩、卡前列素氨丁三醇250 µg宫颈注射，阴道流血明显减少，分娩过程共出血约1 050 ml。6:05患者无明显诱因突发血压下降，监测血压95/54 mmHg（1mmHg = 0.133kPa）、心率116次/min、血氧饱和度95％，患者意识淡漠；同时阴道流血再次增多，出血量约250 ml，考虑不排除羊水栓塞。予甲泼尼龙80 mg静滴、子宫按摩、宫腔球囊填塞等对症治疗仍无好转，送手术室抢救治疗。入术间时患者突发意识丧失、呼吸心跳骤停，予胸外心脏按压、气管插管等急救措施后复苏成功。患者阴道流血持续增多，为不凝血，球囊保守治疗失败，行全子宫切除术+左输卵管切除术+阴道填塞术。术中实验室检查回报：HGB 69 g/L、PLT 48×10^9^/L、凝血酶原时间（PT）33.9 s、活化部分凝血活酶时间（APTT）132 s、纤维蛋白原含量（FIB）0.27 g/L、D-二聚体42 368 µg/L、纤维蛋白原降解产物（FDP）385.2 mg/L、抗凝血酶活性8％，血栓弹力图（thromboelastography，TEG）提示极度低凝合并纤溶亢进。继续补充血液制品，同时予氨甲环酸1 g静滴抗纤溶治疗。术中共出血约5 000 ml，共输注滤白红细胞悬液29 u、滤白病毒灭活冰冻血浆2 800 ml、滤白冷沉淀40 u、血小板1治疗量、纤维蛋白原2.5g、人凝血酶原复合物1 200 IU，术中血压波动（收缩压：70～110 mmHg，舒张压：40～65 mmHg）、心率波动于100～120次/min、尿量1 250 ml。

10:35术毕转入我院重症监护病房（ICU），查体：气管插管呼吸机辅助通气，去甲肾上腺素0.3 µg·kg^−1^·min^−1^维持血压，体温35.4 °C、心率113次/min、血压89/40mmHg、血氧饱和度100％。重度贫血貌、静脉穿刺部位渗血、腹腔引流血性液体、阴道少量流血。实验室检查：HGB 29g/L、PLT 58×10^9^/L、PT 19.0 s、APTT 88 s、FIB 1.07 g/L、D-二聚体5 175 µg/L。予呼吸机辅助通气、补液升压、补充血液制品、氨甲环酸（0.5 g，每日2次）抗纤溶、抗感染、镇静镇痛等治疗。经积极输注血浆、冷沉淀、纤维蛋白原、凝血酶原复合物，抗纤溶及对症支持后，患者凝血功能较前改善，5月8日患者PT 16.8 s、APTT 36 s、FIB 1.30 g/L、D-二聚体2 854 µg/L、FDP 16.5 mg/L。入ICU后共计输注红细胞8 u，HGB仍持续下降至57 g/L，24 h腹腔引流1 080 ml血性液体，考虑存在腹腔内活动性出血，行二次剖腹探查术。术中可见：阴道壁及原子宫切除创面渗血明显，未见明确活动性出血点，行阴道壁缝合止血术+盆腔加固缝合止血+双侧髂内动脉结扎+盆腔纱布压迫填塞术，术中共清除腹腔内积血及血块约3 500 ml，术毕转回ICU。术后复查：HGB 98 g/L、PLT 52×10^9^/L、PT 13.5 s、APTT 36 s、FIB 1.90 g/L、D-二聚体1 230 µg/L，TEG仍提示低凝，予密切监测凝血指标，根据结果回报继续针对性输血。5月9日HGB 94 g/L，术后24 h腹腔引流共300 ml，消毒后取出腹腔内留置油纱。5月13日患者HGB持续稳定，D-二聚体4 365 µg/L，其他凝血指标及TEG结果基本正常，予依诺肝素钠（0.4 ml，每日1次）启动抗凝。5月15日拔除气管插管，7月16日转入我院康复科进一步治疗，11月26日出院。出院时患者神志清楚，生活部分自理，凝血指标等实验室检查未见异常。于我院治疗期间共计输注滤白红细胞悬液56 u、滤白病毒灭活冰冻血浆7 000 ml、滤白冷沉淀120 u、血小板3治疗量、纤维蛋白原9 g、人凝血酶原复合物1 800 IU，无肾功能衰竭等不可逆的器官功能障碍。

## 讨论并文献复习

产科相关DIC是在产科疾病基础上发生的、以出凝血紊乱及微循环障碍为特点的临床病理综合征，羊水栓塞、胎盘早剥、宫内死胎等均为诱发DIC的常见病因[2]，总体发生率为0.03％～0.35％[3]。产科DIC的主要始动环节为组织因子入血，羊水、胎盘及蜕膜等组织中富含的大量组织因子进入母体血液循环，导致凝血级联激活、机体内广泛微血栓形成，引起器官功能损害；凝血物质大量消耗，纤溶系统激活、继发纤溶亢进，临床上通常以血栓形成及出血为突出表现，可危及生命[4]。

早期识别与诊断DIC非常重要。对于广义的DIC诊断，目前已有国际血栓与止血学会（the international society on thrombosis and haemostasis，ISTH）评分、日本急救医学会（the Japanese association for acute medicine，JAAM）DIC标准及中国弥散性血管内凝血诊断积分系统（Chinese DIC scoring system，CDSS）等不同评分系统，各有其优势和局限性，仍无统一的金标准[5]。上述评分系统主要依赖于临床表现及实验室检查，而妊娠期凝血功能的改变可导致凝血指标的生理性变化，故亟需制定针对产妇的DIC诊断标准，同时需注意动态检测、评估相关指标，并与基础值对比以提高诊断效能[6]。2023年发表的《产科弥散性血管内凝血临床诊断与治疗中国专家共识》[4]中的“产科弥散性血管内凝血诊断评分表”可为产科相关DIC的诊断提供参考，本例患者总计评分12分，超过7分，可诊断产科相关DIC。其中产科原发病考虑为羊水栓塞，目前并无国际统一的羊水栓塞诊断标准，仍然是基于临床表现的排除诊断。当产妇在产程中或产后短时间内、突发其他疾病难以解释的低血压、低氧血症、凝血障碍甚至心跳骤停时可考虑诊断[7]，本例患者符合。羊水栓塞诱发的DIC通常进展迅速，表现为显著低凝与异常明显的纤溶亢进[8]，本例亦符合。产科相关DIC需注意与产后出血并发稀释性凝血病相鉴别。两者可有相似的凝血指标改变，均以出血为突出表现，但后者主要由于血液成分的快速大量丢失导致血液稀释，并无DIC病程中的微血栓广泛形成及继发性纤溶亢进。

治疗原发病和纠正凝血功能是治疗产科相关DIC的核心。原发病羊水栓塞的治疗主要以对症器官支持为主[7]，本例患者出现相关症状时已终止妊娠，因此第一时间予大剂量糖皮质激素抗炎抗过敏、气管插管保证充足氧供、应用血管活性药物行循环支持、积极对症输血等治疗。同时产科相关DIC往往合并严重出血或出血倾向，控制出血同样重要。本例患者难治性产后出血保守抢救无效，先后进行2次手术干预及时控制出血，为后续该患者的成功救治提供了基础。

纠正凝血紊乱是治疗DIC的关键环节。典型的DIC病程可分为3个阶段：高凝期、消耗性低凝期、纤溶亢进期。高凝期是DIC发病的起始环节，此时凝血系统激活和广泛微血栓形成，应用抗凝剂从根本上抑制血栓形成对治疗可能是有益的。最常用的药物为肝素，但其主要在DIC早期高凝期使用，存在活动性出血的患者不宜应用[9]。亦有研究指出补充抗凝血酶可能对DIC的治疗有益，但仍缺乏高质量证据[10]。本例患者高凝期极短，难以发现，病情迅速进展至后续的消耗性低凝期，TEG提示极度低凝，已错过抗凝时机；且阴道流出大量不凝血，故并未应用肝素治疗。随着DIC的进展，异常激活的凝血-纤溶系统大量消耗凝血物质，同时大量失血又加剧了血液成分的丢失，造成消耗+稀释性凝血功能障碍，故及时补充血液制品进行替代治疗极为重要，尤其需重视纤维蛋白原的补充[11]。Morikawa等[12]指出，纤维蛋白原水平≤1.5 g/L与产科相关DIC伴出血的发生相关，相应产妇在分娩过程中的失血量明显增加。2023年《产科弥散性血管内凝血临床诊断与治疗中国专家共识》建议[4]，输血维持HGB 70～80 g/L，FIB维持2 g/L以上，根据凝血指标等酌情输注新鲜冰冻血浆及血小板。人凝血酶原复合物可迅速补充凝血因子Ⅱ、Ⅶ、Ⅸ、Ⅹ，但可能增加血栓栓塞风险，在产科相关DIC中的应用尚无足够证据支持，需谨慎使用[13]。本例及时进行了血浆、冷沉淀、纤维蛋白原、血小板等多种血液成分的输注，使得凝血功能及早得到改善，同时快速提高了血液携氧能力，改善组织缺氧。

产科相关DIC可伴有异常明显的纤溶亢进，有研究称在羊水栓塞诱发DIC的病例中，纤维蛋白溶解可能先于凝血功能障碍[14]。纤溶亢进可导致更严重和更广泛的出血，故动态监测纤溶指标、酌情抗纤溶是产科相关DIC治疗的重要部分。TEG可动态完整反映机体凝血与纤溶的全过程，对纤溶状态的判断及血液制品的输注有一定指导意义[15]。氨甲环酸可结合于纤溶酶原的赖氨酸结合位点，抑制纤溶酶原和纤维蛋白结合从而抑制纤溶。一项大型随机、双盲、安慰剂对照试验[16]结果显示，早期给予氨甲环酸可显著降低产后出血患者的死亡率，尤其在产后出血3 h内接受氨甲环酸治疗效果更佳。本例患者在TEG提示纤溶亢进后第一时间加用了氨甲环酸，启动针对性的抗纤溶治疗，并在后续密切复查、调整用药。除纤溶亢进外，还需注意某些少见原因引起的严重产后出血，Wang等[17]报道了1例内源性肝素样物质导致产后出血的病例。该例患者TEG普通杯和TEG肝素酶杯之间的R值存在差异，提示存在肝素活性，而患者并无应用肝素史，故考虑为内源性肝素样物质，予加用鱼精蛋白后患者成功止血。

产科相关DIC患者的综合治疗同样重要。短时间内的大量失血导致组织灌注不足、细胞缺血缺氧，进而导致代谢性酸中毒，同时输血补液过程中大量低温液体的快速输注可能导致低体温，内环境的变化可能抑制凝血酶活性[18]；合并严重感染时组织因子的释放亦会激活凝血导致血栓形成[19]，上述因素均可能加剧凝血障碍的发生，故抗休克、抗感染、体温及内环境管理等亦是DIC救治中的重要环节。血红蛋白水平稳定后尽早应用低分子肝素等预防血栓栓塞性疾病，尤其当凝血过度活化持续存在时，应用抗凝药物可阻止其进展。Wu等[20]报告了一例羊水栓塞诱发DIC的患者，积极治疗1周后HGB显著升高，但多脏器功能不全表现仍突出，凝血指标显示凝血因子和血小板仍被过度激活和消耗，启动抗凝治疗4 d后患者凝血状态得到显著改善，临床症状好转。

总之，在临床中，当产妇出现疑似羊水栓塞、胎盘早剥等情况时，要高度警惕DIC发生，尤其伴有胎盘植入是发生DIC的独立危险因素[21]。发生产科相关DIC时需积极治疗原发病，当存在难以控制的大量出血，需多学科协作，及早评估是否需行外科或介入手术等干预止血；动态监测相关出凝血指标，全面评估凝血状态，针对性、目标性成分输血并酌情抗纤溶。尽快恢复血容量、逆转凝血障碍、提高血液携氧能力[22]，尽最大努力挽救母儿生命。
